# A chemometric study in the area of feasible solution of an acid–base titration of *N*-methyl-6-oxyquinolone

**DOI:** 10.1039/c7ra13427d

**Published:** 2018-03-12

**Authors:** Mathias Sawall, Stella Schmode, Henning Schröder, Ralf Ludwig, Klaus Neymeyr

**Affiliations:** Universität Rostock, Institut für Mathematik Ulmenstraße 69 18057 Rostock Germany klaus.neymeyr@uni-rostock.de; Universität Rostock, Institut für Chemie Dr-Lorenz-Weg 2 18059 Rostock Germany; Leibniz-Institut für Katalyse e.V. an der Universität Rostock Albert-Einstein-Straße 29a 18059 Rostock Germany

## Abstract

Multivariate curve resolution methods aim at recovering the underlying chemical components from spectroscopic data on chemical reaction systems. In most cases the spectra and concentration profiles of the pure components cannot be uniquely determined from the given spectral data. Instead continua of possible factors exist. This fact is known as rotational ambiguity. The sets of all possible pure component factors can be represented in the so-called area of feasible solutions (AFS). This paper presents an AFS study of the pure component reconstruction problem for a series of UV/Vis spectra taken from an acid–base titration of *N*-methyl-6-oxyquinolone. Additional information on the equilibrium concentration profiles for a varying acid concentration is taken from fluorescence measurements. On this basis chemometric duality arguments lead to the construction of a unique final solution.

## Abstract 2

Multivariate curve resolution methods aim at recovering the underlying chemical components from spectroscopic data on chemical reaction systems.

## Graphical abstract

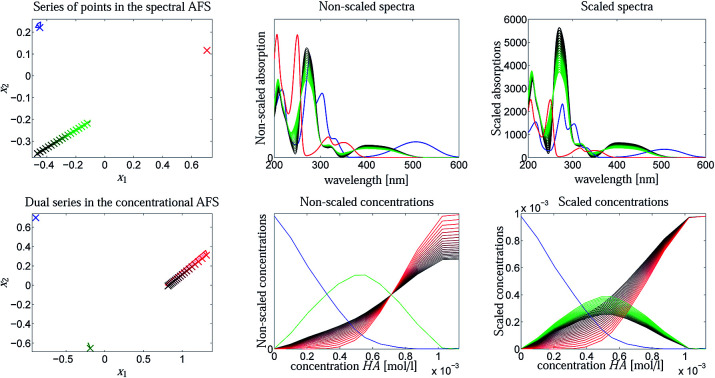

## 1. Introduction

In chemistry and catalysis we are often faced with the problem that the spectral signatures of reactants, intermediates and products overlap. A proper analysis of UV/Vis, fluorescence or infrared spectra as well as deriving kinetics requires a clear model-independent decomposition method. Herein we present a general tool that is based on multivariate curve resolution methods in order to recover pure component spectra and simultaneously the concentration profiles along the reaction coordinate. The concentration profiles can depend on the time (progress of a reaction) or can depend on a changing temperature, acidity and so on. In most cases, a multi-component system cannot be uniquely determined from the given spectra. Mathematically, continua of possible factors exist, including the chemically correct solution. In our method, all possible component factors are represented in the so-called area of feasible solutions (AFS).

Exemplarily, we present an AFS study on the UV/Vis spectra of a recently published dye system, which has only been characterized by a two-component analysis.^1^ The new approach goes much further, which is shown for the titration grades at an acid–base reaction of the dye. Now, systems including more than two components can be decomposed easily. All mathematically possible solutions are displayed in the AFS. With the additional information on the equilibrium concentration profiles for a varying acid concentration taken from fluorescence measurements, the AFS can be reduced to one distinct solution. For the given dye system the concentration profiles have been achieved and the chemical reaction could be described properly.

The AFS approach provides a comfortable graphical user interface and any programming is superfluous. For time dependent measurements reaction kinetics and thermodynamic properties could be derived. Concentration dependent studies such as titrations allow the determination of equilibrium constants, here the acid constant.

### 1.1. Multivariate curve resolution

Multivariate curve resolution (MCR) methods aim at extracting the contributions from the underlying sources to a given data set. An important application in chemometrics is the case that the spectroscopic observation of a chemical reaction system has yielded a matrix *D* ∈ ℝ^*k*×*n*^ of absorption values on a time × frequency grid. Therein *k* is the number of the measured spectra and *n* is the number of spectral channels of each spectrum. The problem is to find the underlying spectra and concentration profiles of the pure components. The Lambert–Beer law in matrix notation relates the pure component recovery problem to the nonnegative matrix factorization problem

1 *D* = *CS*^*T*^.

Proper nonnegative matrix factors *C* ∈ ℝ^*k*×*s*^ and *S* ∈ ℝ^*n*×*s*^ can be interpreted in a way that the *s* columns of *C* are the concentration profiles of the *s* pure components and the columns of *S* are the associated pure component spectra, see *e.g.*ref. 2 and 3. If additional information on the reaction system is available, for example some pure component spectra or concentration profiles, then this can simplify the construction of proper matrix factors *C* and *S*, see *e.g.*ref. 4 and the references therein.

For an overview on chemometric methods for solving the MCR problem see the monographs.^2,3^ The MCR-ALS method^5,6^ is very important. It works with the alternating least squares (ALS). Without claiming any completeness we would also like to mention the window factor analysis,^7^ the evolving factor analysis^8–11^ and the algorithms described in ref. 12 and 13.

Here we focus on MCR methods which use a singular value decomposition (SVD) of the matrix *D*,^2,3,14^ see Sec. 2.1. All these MCR methods suffer from the fact that the nonnegative matrix factorization problem (1) typically has continua of possible solutions (*C*, *S*). This fact is known as “rotational ambiguity” of the solution.^15–19^ Soft-modeling (regularization) or even hard constraints (*e.g.* by kinetic models) are proper tools for reducing the rotational ambiguity, see *e.g.*ref. 2 and 3. In the best case these additional constraints are sufficiently restrictive so that a unique solution can be determined.

An approach for a systematic investigation of the rotational ambiguity is to get access to the set of all nonnegative factorizations in the form^1^ for the given spectral data matrix *D*. A low-dimensional representation of this set is called the area of feasible solutions (AFS), see *e.g.*ref. 16, 17, 20, 21. Within the AFS-setting it is possible to adjoin extra information on the matrix factors, for example by known concentration profiles or spectra, in a very transparent way. By means of duality arguments, see ref. 4, 22–24 this additional information can be used in order to restrict the AFS and to visualize the mutual influence of a given spectrum on the dual concentration profiles and *vice versa*.^25–27^

### 1.2. Contents and organization of the paper

In this paper we analyze series of spectra taken from an acid–base titration of the highly-sensitive dye *N*-methyl-6-oxyquinolone as an acidometer in acetonitrile. First we analyze the ambiguity of the MCR solution. It turns out that considerable ambiguities exist for one spectrum and also for one profile of equilibrium concentrations in dependence on the acid concentration. The application of the so-called closure constraint, namely a mass balance, does not lead to a unique solution. Additional information (namely fixed pure component spectra in combination with fluorescence data) is used in order to construct the final solution. The software FACPACK^17,28^ is used for all computations. The final pure component decomposition is validated against the results of a rank annihilation analysis and a kinetic-model-based factorization;^29,30^ see also the related rank-1 downdates.^31^

The paper is organized as follows: Section 2 introduces SVD-based MCR techniques, the AFS approach for representing the rotational ambiguity and the related duality principles for the solution of the spectral recovery problem. The implementation of these methods in the FACPACK-software is briefly reviewed in Sec. 3. The chemometric analysis for an acid–base titration is contained in Sec. 4.

## 2. Chemometric pure component recovery

Next the AFS and related duality principles are shortly explained. The starting point is the SVD-based construction of factorizations *D* = *CS*^*T*^.

### 2.1. SVD-based construction of pure component factorizations

From a mathematical point of view the factorization (1) is a nonnegative matrix factorization of *D*. Typically, the dimensions *k* and *n* of *D* are much greater than the number of the underlying chemical components *s*. For an appropriate value of *s* (typical values are *s* ≤7) the factors *C* and *S* are computed by means of a truncated SVD of the data matrix.^14^ The truncated SVD has a noise-filtering effect and reads *D* = *UΣV*^*T*^ with orthogonal matrices *U* ∈ ℝ^*k*×*s*^ and *V* ∈ ℝ^*n*×*s*^. Further, *Σ* ∈ ℝ^*s*×*s*^ is a diagonal matrix with the singular values on its diagonal. According to^2,3,14,32^ the factors *C* and *S* can be represented within the truncated bases of left and right singular vectors by means of a basis transformation matrix *T* ∈ ℝ^*s*×*s*^ as follows2
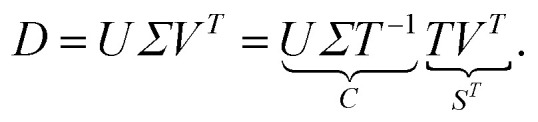


Thus *C* = *UΣT*^−1^ and *S* = *VT*^*T*^ are representations of the *s*(*k* + *n*) matrix elements of *C* and *S* by the much smaller number of *s*^2^ matrix elements of *T* (and its inverse *T*^−1^). Sec. 2.3 shows how these degrees of freedom can be reduced from *s*^2^ to (*s* − 1)*s*. For general *T* the matrices *C* and *S* are called abstract factors and can have large negative entries. The next step is to extract only the nonnegative, chemically relevant factors.

### 2.2. Computation of nonnegative factors

SVD-based MCR methods on the basis of eqn (2) aim at constructing a proper matrix *T* so that *C* and *S* are the chemically correct factors. The matrix *T* can be determined by solving a minimization problem for an objective function which is a weighted combination of penalty/regularization functions.^32–35^ The scalar weight factors enable a proper balance between the different constraints and steer the factorization process. However, the resulting factors *C* and *S* sometimes depend on the constraint presetting of the MCR program. This is an unwanted effect. The minimization of an objective function is usually not sufficient in order to enforce only one, intentionally the chemically correct solution.

In contrast to aiming at a single solution which potentially is only an approximation, it is also possible to compute the sets of all possible nonnegative factors *C* and *S* with *D* = *CS*^*T*^. Such approaches are band boundary computations^36,37^ and the AFS computation.

### 2.3. The area of feasible solutions

The AFS is a low-dimensional representation of either all nonnegative spectra, namely the possible columns of *S*, or all nonnegative concentration profiles, namely the columns of *C*, with *D* = *CS*^*T*^. In other words, we consider all concentration profiles and all spectra which can be extended to nonnegative matrices *C* and *S* in *D* = *CS*^*T*^.^16,17,20,21,38–40^ These feasible columns of *C* or *S* with either *k* or *n* components can be described in a low-dimensional way by the rows of *T*. The reason for this is that the matrix elements of *T* in eqn (2) are the expansion coefficients of the spectra with respect to the basis of the right singular vectors. The associated concentration profiles depend in a similar way on *T*^−1^. Without loss of generality the desired nonnegative spectrum can be assumed to be located in the first column of *S* = *VT*^*T*^, *cf.*eqn (2). The associated expansion coefficients are given by the first row of *T* with the form3
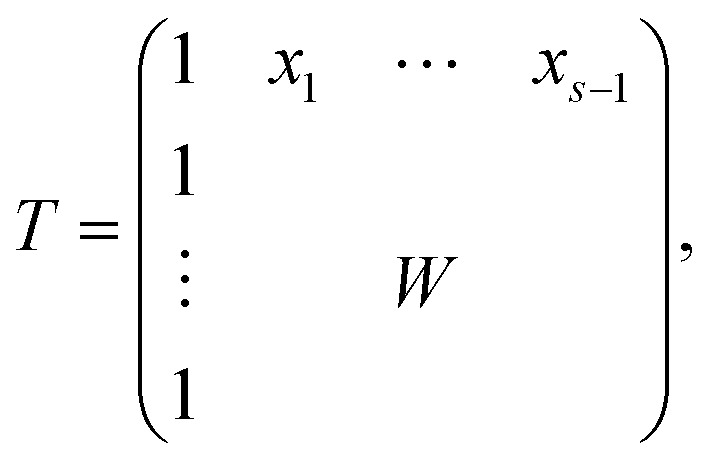
where *W* is an (*s* − 1) × (*s* − 1) submatrix of *T*. The first column of *T* equals the all-ones vector; see ref. 17 for the justification of this implicit scaling. On the basis of these arrangements the AFS for the spectral factor is defined as4
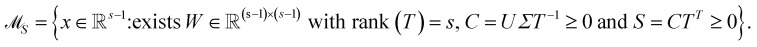


The AFS comprises all (*s* − 1)-dimensional vectors *x* ∈ ℝ^*s*−1^ which can be completed by a matrix *W* ∈ ℝ^(*s*−1)×(*s*−1)^ so that *T* by eqn (3) is a regular matrix and *C*, *S* ≥0. Similarly, one can also define the AFS 
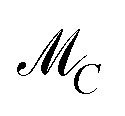
 which represents all feasible nonnegative columns of *C*, see ref. 39.

The AFS sets 
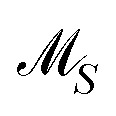
 and 
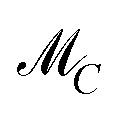
 for two-component systems can easily be constructed.^14,15,41^ Several geometric and numerical algorithms are known to compute the AFS for (*s* = 3)-component systems.^16,17,20,21,28,42–44^ For (*s* = 4)-component systems the AFS computation is much more difficult and only few publications are available.^18,44^ See also ref. 38 and 39 for an overview on the AFS topic.

Here three-component systems (*s* = 3) are in the foci of interest. For this case the polygon inflation method^17,28^ is an effective, very fast and easy-to-control algorithm for AFS computations. In Sec. 3 the software module complementarity & AFS (3 components) of FACPACK is used in order to construct the AFS. It is also used to reduce the ambiguity successively by involving additional system information, see Sec. 2.4.

Up to now we have rigorously assumed nonnegativity of *D*, *C* and *S*. However, experimental spectral data after preprocessing steps, *e.g.* background subtraction, may contain small negative entries. The rank-*s* truncation of the data matrix by the SVD can be a further source of small negative entries. Then small negative entries should also be accepted in *C* and *S* as otherwise the product *CS*^*T*^ cannot reproduce small negative entries of *D*. To this end the polygon inflation algorithm uses a control parameter *ε* ≥0 on the acceptance of small negative entries of *C* and *S*. The feasibility check works as a lower bound on the relative magnitude of negative entries. If rank (*T*) = *s*, then a violation of the inequalities5
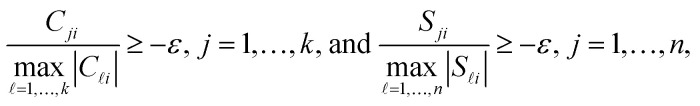
and *i* = 1, …, *s* is used for a penalization in the minimization process.

### 2.4. Duality underlying the factors *C* and *S*

The factorization problem *D* = *CS*^*T*^ is sometimes accompanied by a certain pre-knowledge of parts of the factors. For instance, a spectrum of a reactant or a reaction product might be known or it is possible to determine the concentration profile of a chemical component. A further case is that a frequency window is known in which some of the chemical components are absent.

This information on the columns of *C* and/or *S* can be exploited in order to reduce the rotational ambiguity of the solution. The reason for this is that the constraints of nonnegativity of *C* and *S* and the equality *D* = *CS*^*T*^ imply restrictions on *C* if *S* is partially given and *vice versa*. These mutual constraints are related to the duality principle or complementarity theory.^4,22–24,26^

The underlying idea for the detailed analysis, which is explained in, ref. 4 is based on eqn (2) where *C* and *S* are coupled *via* the matrix *T*. If for example one pure component spectrum is given, then an associated row of *T* can be determined. Due to the equation *T*^−1^*T* = *I*_*s*_, a known row of *T* implies linear and affine constraints on the columns of *T*^−1^. This yields according to *C* = *UΣT*^−1^ in linear, respectively affine, constraints for the columns of *C*. An extreme case is that all but one spectra are given. Then the concentration profile of the remaining/complementary chemical component is uniquely determined except for positive scaling.

### 2.5. Reduction of the AFS by duality arguments

The linear and affine constraints due to known parts of *C* or *S* can be visualized in the AFS.^25–27^ The reduced ambiguity expresses itself in a reduced size of the AFS after taking into consideration the known parts of *C* or *S*. The reduction of the ambiguity is analyzed in this paper for the three-component system of an acid–base titration, see Sec. 4. For this system we demonstrate how a known spectrum of one of the components (this spectrum is represented by a certain point in the AFS) restricts by duality arguments the *s* − 1 concentration profile of the two remaining chemical components. In the AFS of the concentration factor these components are located in an (*s* − 2)-dimensional affine hyperplane. This hyperplane is (in a mathematical sense) dual to a given fixed point in the spectral AFS. To be explicit, the dual affine hyperplane of a three-component system for the case of a given spectrum is a line in the concentrational AFS. Similar relations hold in the reversed direction. For an (*s* = 4)-component system a given point in the spectral AFS is dual to a plane in the concentrational AFS and *vice versa*. See ref. 25 and 45 for more details on these relations and for mathematical formula underlying this duality of points and affine hyperplanes.

## 3. Data analysis with FACPACK

The chemometric analysis in Sec. 4 uses the software package FACPACK which provides a convenient MatLab graphical user interface (GUI) for AFS-computations for two-, three- and four-component systems. The software is available on the FACPACK-homepage.^46^ In particular we utilize the FACPACK module complementarity & AFS (3 components) that serves to construct a pure component decomposition on the basis of the two AFS-sets for the factors *C* and *S*. Known parts of the factors can be identified in the AFS. The program uses duality arguments, see the complementarity theorem,^4^ in order to visualize the correlations of the factors *C* and *S* interactively. This approach reduces the rotational ambiguity of the nonnegative matrix factorization problem drastically.

**Fig. 1:**
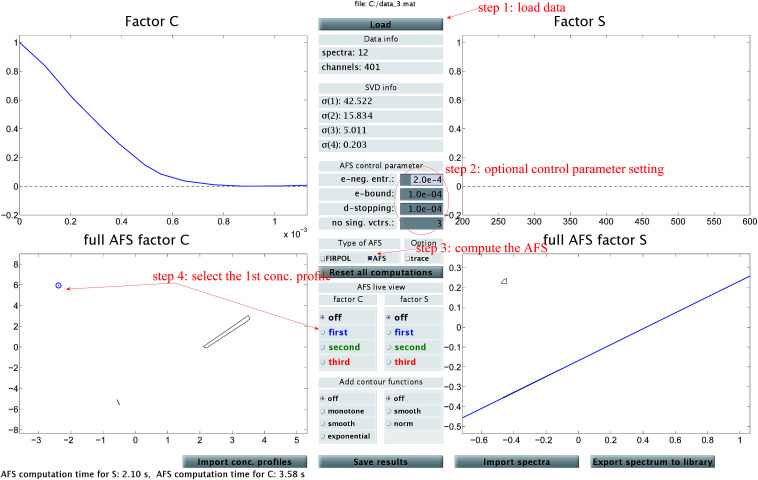
A screen-shot of the graphical user interface of the FACPACK-module complementarity & AFS (3 components). A first concentration profile is constructed. The example data set is explained in Sec. 4. The construction steps are explained in Sec. 3. The boundaries of the two AFS-sets for *C* and *S* are drawn in black in the two lower plots. The user can move the mouse pointer through the AFS and the associated spectrum or concentration profile is shown simultaneously. By pushing the left mouse button, a certain solution can be fixed. The different scaling in the plot of 
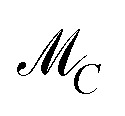
 compared to the AFS plots in Fig. 6–8 is explained by the fact that the matrix *Σ* is taken into account here, but is omitted in Fig. 6–8.

**Fig. 2:**
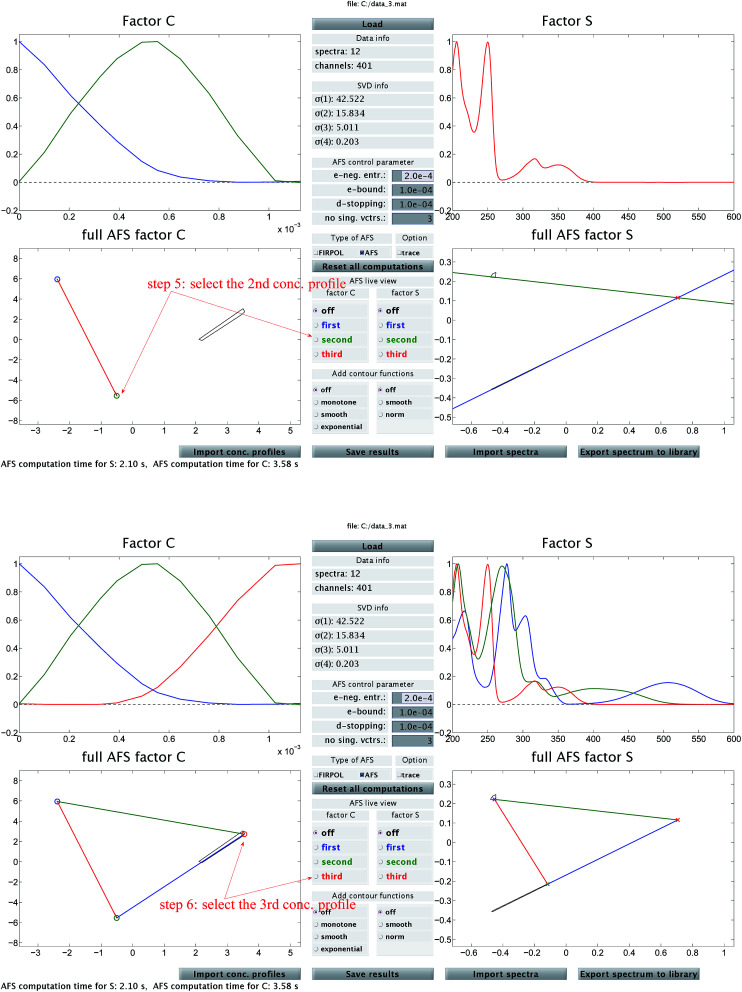
In addition to Fig. 1 these two screen-shots demonstrate the construction of the second (upper screen-shot) and of the third (lower screen-shot) concentration profile. The duality theory increasingly limits the feasible solutions, which means that the rotational ambiguity is reduced.

The steps of the chemometric analysis are illustrated by Fig. 1 and 2 that show screen-shots of this program if applied to the UV/Vis-data of Sec. 4. First the spectral data is loaded to the program (see step 1 in Fig. 1). Certain control parameters can be set in an optional step (see step 2 in Fig. 1). The AFS sets are drawn after checking the AFS box (see step 3). The chemometric pure component reconstruction is started by selecting the radio button first (see step 4). Then the mouse pointer can be moved through the concentrational AFS. Simultaneously the concentration profile which belongs to the AFS-coordinates under the mouse pointer is drawn. Any solution can be locked by clicking the left mouse button. The selected solution in the concentrational AFS is linked to a straight line in the spectral AFS (by duality arguments). This blue straight line in Fig. 1 represents a significant restriction on the feasible spectral profiles.

Then Fig. 2 (upper screen shot) demonstrates how a second concentration profile is determined. Once again, duality arguments result in restrictions in the spectral AFS, see the green straight line. The point of intersection of these two straight lines uniquely determines the spectrum of one chemical component. Finally, the screen shot in the lower part of Fig. 2 illustrates how the pure component decomposition is completed after determining a third concentration profile. The user has then the option to refine the decomposition by releasing any arbitrary concentration or spectral profiles and to modify it until a complete optimal solution is found.

The FACPACK software uses the polygon inflation algorithm for AFS computations and provides all the chemometric software tools within a conveniently usable graphical user interface. This includes interfaces for the data import, for an optional data preprocessing and the data export. Other AFS computation methods are the so-called Borgen plots^20,21^ and the recent dual Borgen plot approach.^45,47^ Alternatively, the rotational ambiguity underlying MCR factorizations can be illustrated in terms of the bands of feasible profiles^36,37^ and by using the MCR-Bands software. The steps of our chemometric analysis can be applied in similar form to the sets of feasible bands.

### 3.1. Control parameter setting

The numerical AFS computation is controlled by several parameters, *e.g.* stopping criteria for the optimization procedure, the boundary precision, a bound on the sum of least squares of the objective function, the maximal number of cycles of the optimization and the maximal number of function evaluations. For the detailed description of these parameters see ref. 17. The program provides default values for all parameters which ensure in most cases a stable, precise and fast AFS computation. Finally, the parameter *ε* in eqn (5) controls the size of acceptable negative entries of *C* and *S* and thus the size of the AFS. Increasing *ε* results in an expansion of the AFS-sets. For all computations we used *ε* = 2 × 10^−4^.

## 4. Chemometric analysis of an acid–base titration

**Fig. 3:**
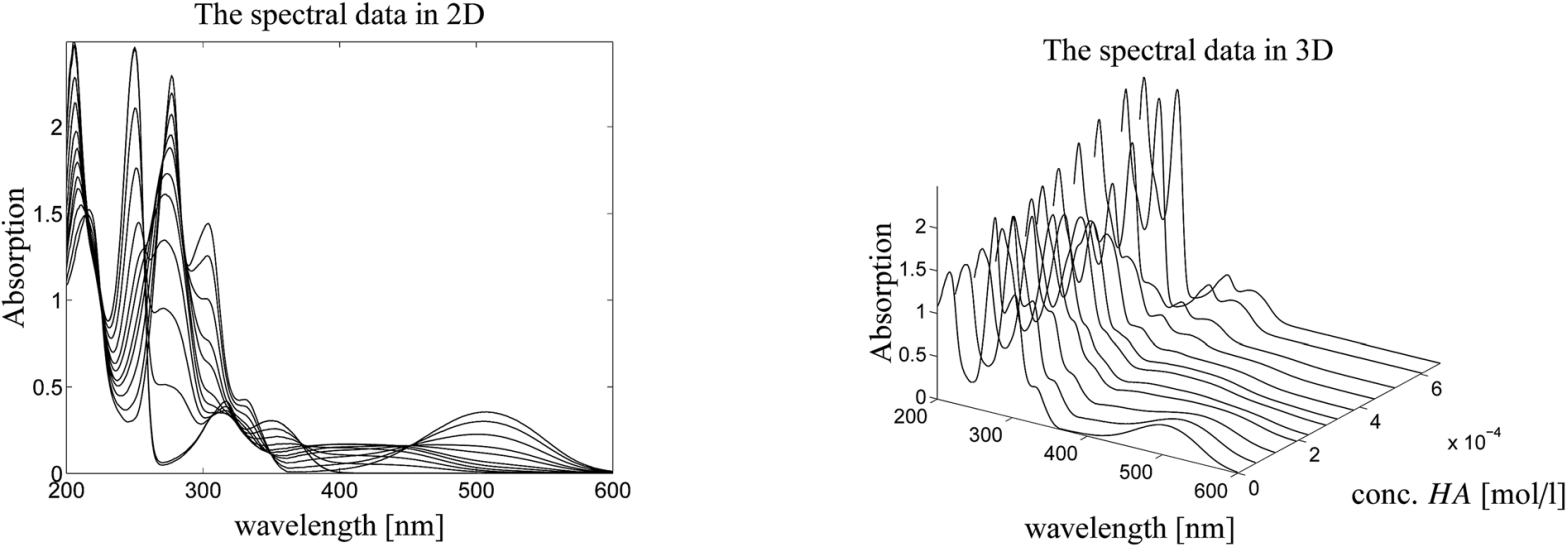
Series of UV/Vis spectra on the protonation of MQz in acetonitrile. Left: 2D-plot. Right: 3D-plot.

Here we study a series of UV/Vis spectra of a titration of *N*-methyl-6-oxyquinolone (MQz) in acetonitrile with the trifluoromethanesulfonic superacid. The acid is denoted by HA. The series of spectra is plotted in Fig. 3. The AFS is constructed for the spectral factor and for the factor of equilibrium concentration profiles in dependence on the acid concentration. Finally, a unique pure component factorization is constructed by involving information on known pure component spectra and fluorescence measurements of the equilibrium concentrations. The addition of information for the two matrix factors *C* and *S* distinguishes the present approach from other works as.^25–27^ See Sec. 4.4 for the details.

### 4.1. Experiment and spectral data

**Fig. 4:**
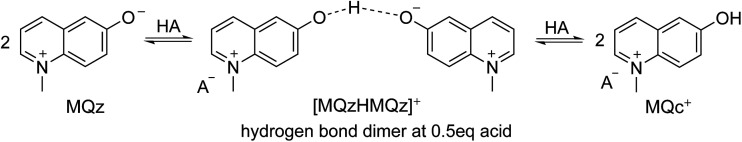
Reaction scheme of the proton transfer to *N*-methyl-6-oxyquinolone (MQz), dimerization to [MQzHMQz]^+^ and split of the dimer to MQc^+^ with an increasing acid concentration.

Fig. 4 shows the protonation scheme of *N*-methyl-6-oxyquinolone (MQz) which includes an intermediate dimerization, see also ref. 48. A total number of *k* = 12 UV/Vis spectra are taken for increasing concentration values of the superacid HA. The interval of concentration values of HA is [0, 1.264 × 10^−3^]mol l^−1^. Each spectrum is a vector with *n* = 401 components which are the absorption values in the wavelength window [200, 600]nm. Hence, *D* ∈ ℝ^12×401^. Fig. 3 shows the series of spectra in a 2D- and a 3D-plot.

The three dominant chemical components of this reaction system are the chemical indicator MQz, the dimer species [MQzHMQz]^+^, the protonated indicator MQc^+^ as well as HA and A^−^. The latter two components in negligible extent contribute to the absorption in the analyzed wavelength interval. The reaction equations with kinetic constants read6
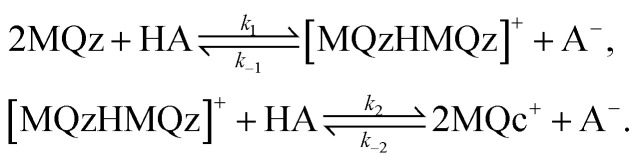


For stoichiometric reasons the weighted sum of concentration values fulfills

7 *c*(MQz) + 2*c*([MQzHMQz]^+^) + *c*(MQc^+^) = *c*_0_

with the initial concentration *c*_0_ = 9.84269 × 10^−4^ mol l^−1^. The chemometric analysis is based on the following steps: first we compute an SVD of *D* and also the AFS sets, see Sec. 4.2. The rotational ambiguity which is represented by these AFS sets is then visualized in terms of feasible bands, see Sec. 4.3. In order to reduce the rotational ambiguity, we add in a first step the pure component spectrum of the reactant MQz and in a second step the equilibrium concentration profiles of MQz and MQc^+^, see Sec. 4.4.

### 4.2. SVD and AFS computation

**Fig. 5:**
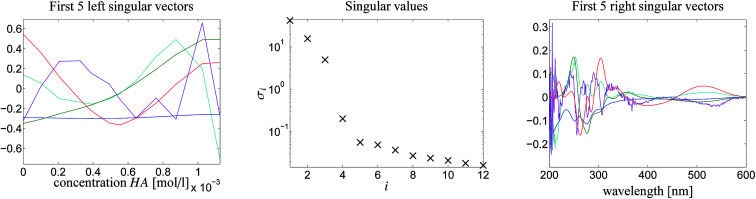
The first 5 left/right singular vectors and the singular values in a semi-logarithmic plot. The SVD indicates the existence of three dominant absorbing components. Colors of singular vectors: blue (1), green (2), red (3), cyan (4) and purple (5).

Fig. 5 shows the first five left/right singular vectors and the 12 singular values of *D*. These data clearly indicate three dominant singular values and thus only MQz, [MQzHMQz]^+^ and MQc^+^ have relevant absorptions in the given wavelength window. This result is confirmed by the associated three left/right singular vectors which have a non-oscillatory character and are expected to include relevant structural information. The singular values and the singular vectors indicate a relatively large signal-to-noise ratio for the given spectra *D*. This is a good basis for a successful construction of the two AFS sets and also for exploiting the underlying duality of the factors *C* and *S*. The polygon inflation method is applied with *δ* = *ε*_b_ = 10^−4^ and *ε* = 2 × 10^−4^ as upper bounds on the relative size of negative entries.

**Fig. 6:**
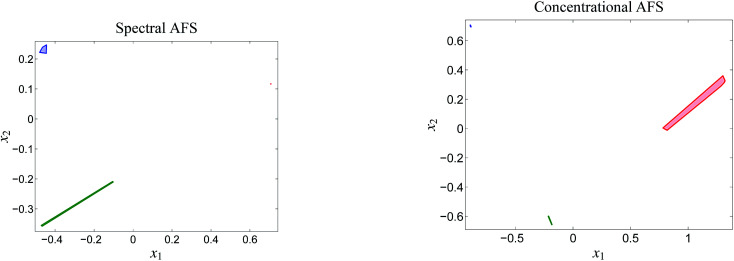
The two AFS-sets for the acid–base titration data. Each of the AFS-sets consists of three isolated subsets which represent the three chemical components MQz (blue), [MQzHMQz]^+^ (green) and MQc^+^ (red). The results are computed with *ε* = 2 × 10^−4^.

The AFS-sets indicate a small ambiguity of the solution for the two components MQz and MQc^+^ (in blue and red) in the spectral AFS since the area of the associated subsets of the AFS is very small. The subsets of the concentrational AFS which belong to the components MQz (blue) and [MQzHMQz]^+^ (green) are also small. Thus the associated series of spectra and concentration profiles only show a small variation. In other words the rotational ambiguity is of moderate magnitude. Only the pure component spectrum of [MQzHMQz]^+^ and concentration profile of MQc^+^ contain considerable ambiguities.

### 4.3. Bands of possible profiles representing the ambiguity

**Fig. 7:**
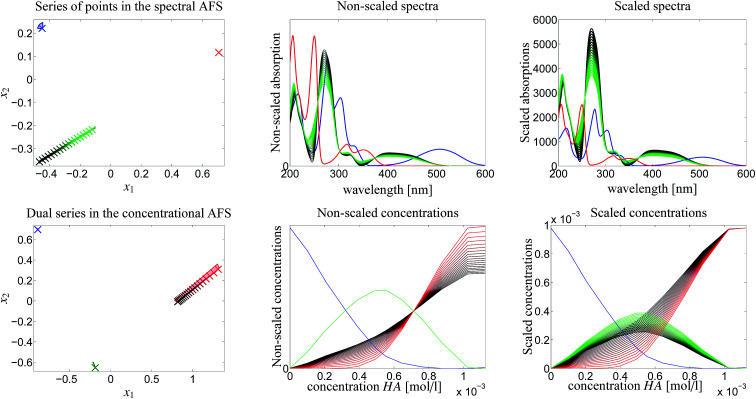
AFS-based analysis of the rotational ambiguity. Color code: MQz in blue color, [MQzHMQz]^+^ in green color and MQc^+^ in red color. Left column of plots: in the spectral AFS two spectra (crosses for MQz and MQc^+^) are fixed due to their low ambiguity. A series of points in the green subset of the AFS is considered. These are marked by green crosses and represent a series of possible spectra of [MQzHMQz]^+^. By duality arguments the equilibrium concentration profile of [MQzHMQz]^+^ is uniquely determined, see the green cross in the concentrational AFS. The blue crosses (MQz) only show a small variability, whereas the equilibrium concentration profiles of MQc^+^ (red) show a strong variation. The remaining four subplots show the bands of spectra and concentration profiles which belong to the marked points in the AFS. The width of these bands is large if the points in the AFS show a strong variation. These plots show the profiles in a non-scaled and also in a scaled form; see the explanations.

The rotational ambiguity inherent to an AFS can also be represented by drawing the associated bands of feasible spectra and the band of feasible equilibrium concentration profiles. This is done in Fig. 7. The colored crosses in the left two AFS plots mark positions for which the associated spectra or concentration profiles are drawn. More than one point for one chemical component is considered in the spectral AFS of [MQzHMQz]^+^ and in the concentrational AFS of MQc^+^.

The series of spectra and concentration profiles are drawn in Fig. 7. The upper row of plots show the spectral AFS and their spectral bands. The color code for the AFS sets and the bands is as follows. Blue color is used for MQz, green for [MQzHMQz]^+^ and red for MQc^+^. The subsets of the AFS-sets with the largest area, namely [MQzHMQz]^+^ in the spectral AFS and MQc^+^ in the concentrational AFS, are associated to the series of the feasible spectra (green) and concentration profiles (red), see the centered column of Fig. 7.

The two plots in the centered column of Fig. 7 show the bands of the possible factors in a non-scaled form (as obtained by the FACPACK software). Two spectra (MQz and MQc^+^) and one concentration profile ([MQzHMQz]^+^) are almost uniquely determined; the latter by duality. The equilibrium concentration profile of (MQz) has a very low rotational ambiguity. However, the spectrum of [MQzHMQz]^+^ and the concentration profile of MQc^+^ show a considerable ambiguity.

The two plots in the right column of Fig. 7 show the same profiles after an application of a scaling with respect to the so-called closure constraint, which is the mass balance underlying.^7^ The scaling constants are computed in the sense of least-squares along the full acid concentration axis. This results in concentration values of MQc^+^ equal to the initial value *c*_0_ = 9.84269 × 10^−4^ at the highest acid concentration. A side effect of this scaling is that an additional scaling ambiguity appears for the concentration profile of the dimer [MQzHMQz]^+^ (green curves). In other words the profile of this component has been qualitatively determined, but not quantitatively. With the given information on the system this remaining ambiguity cannot be broken up. For the related triples of concentration profiles in the right lower plot of Fig. 7 the squared sum of errors
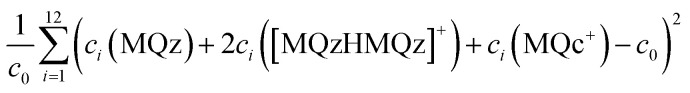
has approximately the value 4.1 × 10^−8^. Therein the index *i* runs through the 12 different values of the acid concentration for which the equilibrium concentrations of the three components MQz, [MQzHMQz]^+^ and MQc^+^ are to be determined.

### 4.4. Involvement of additional chemometric information

**Fig. 8:**
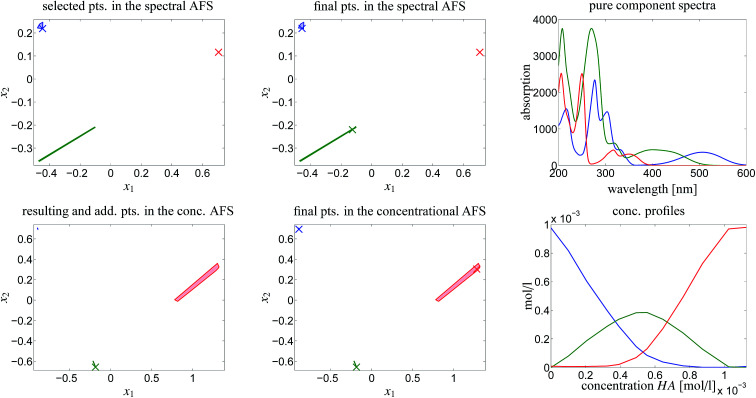
Reconstruction of the final solution as explained in Sec. 4.4. Upper left plot: two pure component spectra are fixed in the spectral AFS (× markers). Lower left plot: the duality underlying *C* and *S* in *D* = *CS*^*T*^ uniquely determines one point in the concentrational AFS (green × marker). Centered column of figures: fluorescence measurements determine two of the concentration profiles (blue and red markers in the concentrational AFS). Once again a duality argument uniquely determines the spectrum of the dual spectrum, namely the spectrum of the dimer [MQzHMQz]^+^. Right column of figures: the final pure component factorization.

In order to attain a final and unique pure component decomposition some additional information on the chemical reaction system is to be added. This is done in two steps:

First the pure component spectrum of MQz is set to be equal to the first measured spectrum *D*(1,:). The justification for this is that the concentration vector of the three chemical components for an initial acid concentration of zero equals (*c*_0_,0,0). Furthermore, the last spectrum *D*(12,:) is set to the pure component spectrum of component MQc^+^. This fixes two points in the spectral AFS. The underlying duality uniquely determines (up to scaling) the equilibrium concentration profile of the dimer [MQzHMQz]^+^, see the left column of plots in Fig. 8. As explained in Sec. 4.3 some ambiguity still remains.

The second step is that fluorescence measurements make it possible to determine the equilibrium concentration profiles of MQz (blue curve) and MQc^+^ (red curve). Once again the duality of these known parts of the factor *C* to the factor *S* uniquely determines the spectrum of the dimer [MQzHMQz]^+^. This completes the pure component recovery. All results are shown in Fig. 8.

### 4.5. Result verification by means of rank annihilation and kinetic-hard modeling

In Sec. 4.4 we have involved the pure component spectra of MQz and of MQc^+^ to the final pure component recovery. Good approximations of these spectra are accessible from the first and last column of *D*. The associated concentration values are *C*(1) = (*c*_0_,0,0) and *C*(12,:) = (0,0,*c*_0_). These data also make it possible to apply a rank annihilation analysis^29,30^ in the form of two rank-1 downdates^31^8
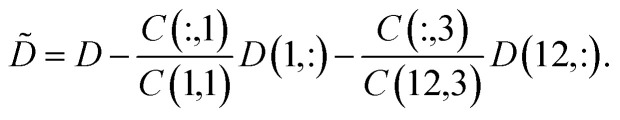


**Fig. 9:**
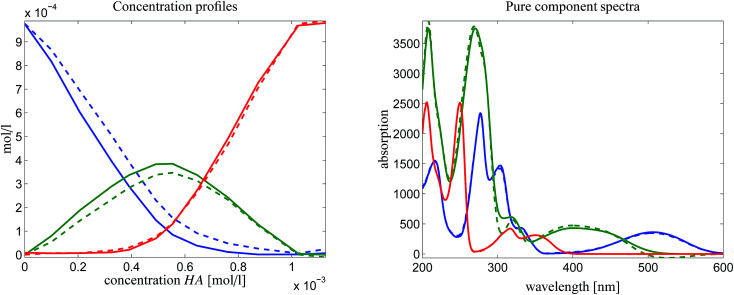
Comparison of the results of a rank annihilation analysis (dashed lines) with the results of the AFS-based approach for the reduction of the rotational ambiguity.

If perturbations are ignored, then *D̃* is a rank-1 matrix which contains in its columns only multiples of the spectrum of the dimer [MQzHMQz]^+^. For experimental spectral data we must take into account noise and other perturbations. Thus a singular value decomposition of *D̃* is applied. The left and the right singular vectors corresponding to the largest singular value are the desired equilibrium concentration profile and spectrum of [MQzHMQz]^+^. The profiles are plotted in Fig. 9 by dashed lines. The results of the AFS-based approach are plotted by solid lines. Relevant difference must be stated in particular for the spectrum of the dimer [MQzHMQz]^+^ which attains close to 500 nm a minimal negative component of −1.7 × 10^−2^ by rank annihilation. The AFS-based approach prevents negative entries of such a magnitude. There are also differences between the equilibrium concentration profiles of the two methods.

**Fig. 10:**
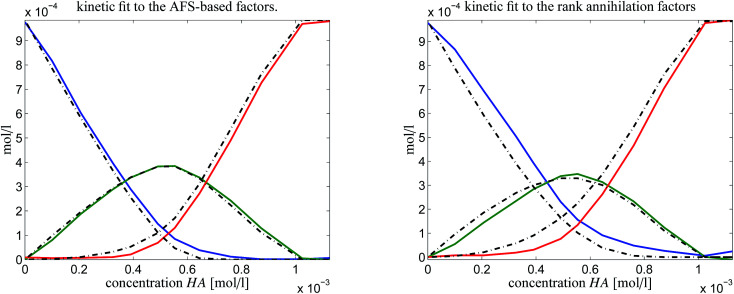
Kinetic model fits (dash-dotted lines) to the two solutions as shown in Fig. 9.

In order to judge which of the approaches provides the better results, we have fitted the kinetic model eqn (6) to the computed pure component factors each for the two computational approaches. Such kinetic models are well known to be stringent decision makers.^40^ For these computations we have set *k*_−1_ = *k*_−2_ = 0 as the trifluoromethanesulfonic superacid does not let expect a notable back reaction. The results are plotted in Fig. 10. They clearly indicate that the AFS-based decomposition provides the better results. This conclusion is supported by the following relative error values
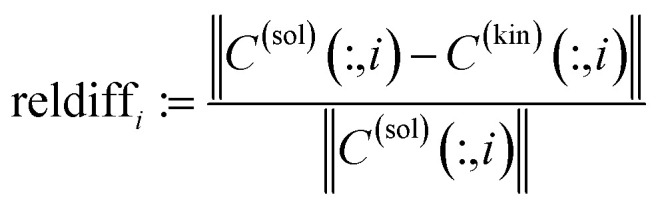
on the differences of the kinetic-model-based concentration profiles *C*^(kin)^(:,*i*) for the components for *i* = 1, 2, 3 to the solution profiles *C*^(sol)^(:,*i*) of the AFS-based approach and the rank annihilation approach. These relative errors have been computed with respect to the maximum norm (maximal value of absolute error values) and the Euclidean norm (sum of squares)AFS-based solution: ‖·‖_max_: reldiff = (0.039, 0.050, 0.048), ‖·‖_2_: reldiff = (0.089, 0.088, 0.103),Rank annihilation: ‖·‖_max_: reldiff = (0.117, 0.121, 0.092), ‖·‖_2_: reldiff = (0.235, 0.223, 0.195).

## 5. Conclusion

The ambiguity of the solutions of the pure component factorization problem is a fundamental complication, which is often hidden by the fact that MCR software packages produce only one solution. However, this single solution must be considered to be only a more or less reliable approximation of the true solution. In this study we have shown that a unique pure component decomposition can be gained for the given three-component system consisting of *N*-methyl-6-oxyquinolone (MQz), the zwitterionic species [MQzHMQz]^+^ and MQc^+^. The underlying rotational ambiguity of the pure component factorization problem for this system is computed and represented in the AFS. *Versus* the background of the AFS, various chemometric techniques are employed in order to reduce the ambiguity. The final pure component factorization is verified against an alternative chemometric approach and also against a kinetic-model of the reaction scheme. The results underline the effectiveness of AFS-based chemometric analyses and demonstrates the effectiveness of MQz as an optical acidometer.

## Conflicts of interest

There are no conflicts to declare.

## Supplementary Material

## References

[cit1] Schmode S., Ludwig R. (2017). Chem. Commun..

[cit2] Malinowski E. , Factor analysis in chemistry, Wiley, New York, 2002

[cit3] Maeder M. and Neuhold Y., Practical data analysis in chemistry, Elsevier, Amsterdam, 2007

[cit4] Sawall M., Fischer C., Heller D., Neymeyr K. (2012). J. Chemom..

[cit5] Jaumot J., Gargallo R., de Juan A., Tauler R. (2005). Chemom. Intell. Lab. Syst..

[cit6] Jaumot J., de Juan A., Tauler R. (2015). Chemom. Intell. Lab. Syst..

[cit7] Malinowski E. (1992). J. Chemom..

[cit8] Maeder M., Zuberbühler A. D. (1986). Anal. Chim. Acta.

[cit9] Maeder M. (1987). Anal. Chem..

[cit10] Maeder M., Zilian A. (1988). Chemom. Intell. Lab. Syst..

[cit11] Keller H., Massart D. (1991). Chemom. Intell. Lab. Syst..

[cit12] Lee D., Seung H. (1999). Nature.

[cit13] Kim H., Park H. (2008). SIAM Journal on Matrix Analysis and Applications.

[cit14] Lawton W., Sylvestre E. (1971). Technometrics.

[cit15] Abdollahi H., Tauler R. (2011). Chemom. Intell. Lab. Syst..

[cit16] Golshan A., Abdollahi H., Maeder M. (2011). Anal. Chem..

[cit17] Sawall M., Kubis C., Selent D., Börner A., Neymeyr K. (2013). J. Chemom..

[cit18] Golshan A., Maeder M., Abdollahi H. (2013). Anal. Chim. Acta.

[cit19] Zhang X., Tauler R. (2015). Chemom. Intell. Lab. Syst..

[cit20] Borgen O., Kowalski B. (1985). Anal. Chim. Acta.

[cit21] Rajkó R., István K. (2005). J. Chemom..

[cit22] Henry R. (2005). Chemom. Intell. Lab. Syst..

[cit23] Rajkó R. (2006). J. Chemom..

[cit24] Neymeyr K., Sawall M. (2016). J. Chemom..

[cit25] Sawall M., Neymeyr K. (2014). Anal. Chim. Acta.

[cit26] Beyramysoltan S., Abdollahi H., Rajkó R. (2014). Anal. Chim. Acta.

[cit27] Hemmateenejad B., Shojaeifard Z., Shamsipur M., Neymeyr K., Sawall M., Mohajeri A. (2015). RSC Adv..

[cit28] Sawall M., Neymeyr K. (2014). J. Chemom..

[cit29] Ho C.-N., Christian G., Davidson E. (1978). Anal. Chem..

[cit30] Abdollahi H., Nazari F. (2003). Anal. Chim. Acta.

[cit31] Biggs M. , Ghodsi A. and Vavasis S., Proceedings of the 25th International Conference on Machine Learning, New York, NY, USA, 2008, pp. 64–71

[cit32] Neymeyr K., Sawall M., Hess D. (2010). J. Chemom..

[cit33] Haario H., Taavitsainen V. (1998). Chemom. Intell. Lab. Syst..

[cit34] de Juan A., Maeder M., Martínez M., Tauler R. (2000). Chemom. Intell. Lab. Syst..

[cit35] Widjaja E., Li C., Chew W., Garland M. (2003). Anal. Chem..

[cit36] Gemperline P. (1999). Anal. Chem..

[cit37] Tauler R. (2001). J. Chemom..

[cit38] Golshan A., Abdollahi H., Beyramysoltan S., Maeder M., Neymeyr K., Rajkó R., Sawall M., Tauler R. (2016). Anal. Chim. Acta.

[cit39] Sawall M. , Jürß A., Schröder H. and Neymeyr K., On the analysis and computation of the area of feasible solutions for two-, three- and four-component systems, in vol. 30 of Data Handling in Science and Technology, “Resolving Spectral Mixtures”, ed. C. Ruckebusch, Elsevier, Cambridge, 2016, ch. 5, pp. 135–184

[cit40] Schröder H., Sawall M., Kubis C., Selent D., Hess D., Franke R., Börner A., Neymeyr K. (2016). Anal. Chim. Acta.

[cit41] Abdollahi H., Maeder M., Tauler R. (2009). Anal. Chem..

[cit42] Jürß A., Sawall M., Neymeyr K. (2015). J. Chemom..

[cit43] Ghaheri S., Masoum S., Gholami A. (2016). J. Chromatogr. A.

[cit44] Sawall M., Neymeyr K. (2017). Anal. Chim. Acta.

[cit45] Sawall M., Jürß A., Schröder H., Neymeyr K. (2017). J. Chemom..

[cit46] Sawall M. , Jürß A. and Neymeyr K., FACPACK: A software for the computation of multi-component factorizations and the area of feasible solutions, Revision 1.3, FACPACK homepage, http://www.math.uni-rostock.de/facpack/, 2015

[cit47] Sawall M., Moog A., Kubis C., Schröder H., Selent D., Franke R., Brächer A., Börner A., Neymeyr K. (2018). J. Chemom..

[cit48] Pérez-Lustres J., Rodriguez-Prieto F., Mosquera M., Senyushkina T., Ernsting N., Kovalenko S. (2007). J. Am. Chem. Soc..

